# Peroxisome proliferator-activated receptor γ1 expression is diminished in human osteoarthritic cartilage and is downregulated by interleukin-1β in articular chondrocytes

**DOI:** 10.1186/ar2151

**Published:** 2007-03-26

**Authors:** Hassan Afif, Mohamed Benderdour, Leandra Mfuna-Endam, Johanne Martel-Pelletier, Jean-Pierre Pelletier, Nicholas Duval, Hassan Fahmi

**Affiliations:** 1 Osteoarthritis Research Unit, Centre Hospitalier de l'Université de Montréal (CHUM), Notre-Dame Hospital, Department of Medicine, University of Montreal, Montreal, 1560 Sherbrooke East, Pavillon J.A DeSève, Y-2628, Montreal, QC, H2L 4M1, Canada; 2 Centre de Recherche, Sacré-Coeur Hospital, 5400 Boulevard Gouin Ouest, Montréal, QC, H4J 1C5, Canada; 3 Centre de Convalescence, Pavillon de Charmilles, 1487 Boulevard des Laurentides, Montréal, QC, H7M 2Y3, Canada

## Abstract

Peroxisome proliferator-activated receptor γ (PPARγ) is a nuclear receptor involved in the regulation of many cellular processes. We and others have previously shown that PPARγ activators display anti-inflammatory and chondroprotective properties *in vitro *and improve the clinical course and histopathological features in an experimental animal model of osteoarthritis (OA). However, the expression and regulation of PPARγ expression in cartilage are poorly defined. This study was undertaken to investigate the quantitative expression and distribution of PPARγ in normal and OA cartilage and to evaluate the effect of IL-1β, a prominent cytokine in OA, on PPARγ expression in cultured chondrocytes. Immunohistochemical analysis revealed that the levels of PPARγ protein expression were significantly lower in OA cartilage than in normal cartilage. Using real-time RT-PCR, we demonstrated that PPARγ1 mRNA levels were about 10-fold higher than PPARγ2 mRNA levels, and that only PPARγ1 was differentially expressed: its levels in OA cartilage was 2.4-fold lower than in normal cartilage (*p *< 0.001). IL-1 treatment of OA chondrocytes downregulated PPARγ1 expression in a dose- and time-dependent manner. This effect probably occurred at the transcriptional level, because IL-1 decreases both PPARγ1 mRNA expression and PPARγ1 promoter activity. TNF-α, IL-17, and prostaglandin E_2 _(PGE_2_), which are involved in the pathogenesis of OA, also downregulated PPARγ1 expression. Specific inhibitors of the mitogen-activated protein kinases (MAPKs) p38 (SB203580) and c-Jun N-terminal kinase (SP600125), but not of extracellular signal-regulated kinase (PD98059), prevented IL-1-induced downregulation of PPARγ1 expression. Similarly, inhibitors of NF-κB signaling (pyrrolidine dithiocarbamate, MG-132, and SN-50) abolished the suppressive effect of IL-1. Thus, our study demonstrated that PPARγ1 is downregulated in OA cartilage. The pro-inflammatory cytokine IL-1 may be responsible for this downregulation via a mechanism involving activation of the MAPKs (p38 and JNK) and NF-κB signaling pathways. The IL-1-induced downregulation of PPARγ expression might be a new and additional important process by which IL-1 promotes articular inflammation and cartilage degradation.

## Introduction

Osteoarthritis (OA) is the most common joint disorder, accounting for a large proportion of disability in adults. It is characterized by the progressive destruction of articular cartilage, and excessive production of several pro-inflammatory mediators [1-3]. Among these mediators, IL-1β has been shown to be predominantly involved in the initiation and progression of the disease [1-3]. Exposure of chondrocytes to IL-1 induces a cascade of inflammatory and catabolic events including the upregulation of genes encoding matrix metalloproteinases (MMPs), aggrecanases, inducible nitric oxide synthase, cyclooxygenase-2 (COX-2), and microsomal prostaglandin E synthase-1 (mPGES-1) [1-4], leading to articular inflammation and destruction. Although the role of increased inflammatory and catabolic responses in OA is well documented, little is known about the endogenous signals and pathways that negatively regulate these events. Thus, identification and characterization of these pathways is of major importance in improving our understanding of the pathogenesis of OA and may be helpful in the development of new efficacious therapeutic strategies.

Peroxisome proliferator-activated receptors (PPARs) are a family of ligand-activated transcription factors belonging to the nuclear receptor superfamily [5]. So far, three PPAR subtypes have been identified: PPARα, PPARβ/δ, and PPARγ. PPARα is present mostly in the liver, heart, and muscle, where it is the target of the fibrate class of drugs and is believed to function in the catabolism of fatty acid [6]. PPARβ/δ is fairly ubiquitous and seems to be important in lipid and energy homeostasis [7]. PPARγ is the most studied form of PPAR. At least two PPARγ isoforms have been identified that are derived from the same gene by the use of alternative promoters and differential mRNA splicing [8,9]. PPARγ1 is found in a broad range of tissues, whereas PPARγ2 is expressed mainly in adipose tissue [10].

Several lines of evidence suggest that PPARγ activation may have therapeutic benefits in OA and possibly other chronic articular diseases. We and others have shown that PPARγ is expressed and functionally active in chondrocytes and that PPARγ activators modulate the expression of several genes considered essential in the pathogenesis of OA. PPARγ activation inhibits the IL-1-induced expression of inducible nitric oxide synthase, MMP-13, COX-2, and mPGES-1 in chondrocytes [4,11,12]. Moreover, pretreatment with PPARγ activators prevents IL-1-induced proteoglycan degradation [13]. Additionally, PPARγ activation in synovial fibroblasts prevents the expression of IL-1, TNF-α, MMP-1, COX-2, and mPGES-1 [14-16]. The inhibitory effect of PPARγ is partly due to antagonizing the transcriptional activity of the transcription factors NF-κB, activator protein 1 (AP-1), signal transducers and activators of transcription (STATs), and Egr-1 [16,17]. The protective effect of PPARγ activators has also been demonstrated in several animal models of arthritis, including a guinea-pig model of OA [18]. In that study, pioglitazone, a PPARγ activator, reduced cartilage degradation as well as IL-1 and MMP-13 expression [18]. Together, these data indicate that PPARγ may constitute a new therapeutic target in treating OA.

Although a considerable amount is known on the effects of PPARγ activation on inflammatory and catabolic responses in articular tissues, little is known about PPARγ expression and regulation in these tissues. To improve our understanding of the biology of PPARγ in OA, we compared the expression of PPARγ in normal and OA cartilage. In addition, we investigated the effect of IL-1 on PPARγ expression in human OA chondrocytes.

## Materials and methods

### Reagents

Recombinant human IL-1β was obtained from Genzyme (Cambridge, MA, USA), and recombinant human TNF-α and recombinant human IL-17 were from R&D Systems (Minneapolis, MN, USA). Prostaglandin E_2 _(PGE_2_) was from Cayman Chemical Co. (Ann Arbor, MI, USA). SB203580, SP600125, PD98059, pyrrolidine dithiocarbamate (PDTC), MG-132 and SN-50 were from Calbiochem (La Jolla, CA, USA). DMEM, penicillin and streptomycin, FCS, and TRIzol^® ^reagent were from Invitrogen (Burlington, ON, Canada). All other chemicals were purchased from either Bio-Rad (Mississauga, ON, Canada) or Sigma-Aldrich Canada (Oakville, ON, Canada).

### Specimen selection and chondrocyte culture

Human normal cartilage (from femoral chondyles) was obtained at necropsy, within 12 hours of death, from donors with no history of arthritic diseases (*n *= 18, age 61 ± 15 years (mean ± SD)). To ensure that only normal tissue was used, cartilage specimens were thoroughly examined both macroscopically and microscopically. Only those with neither alteration were processed further. Human OA cartilage was obtained from patients undergoing total knee replacement (*n *= 41, age 64 ± 14 years (mean ± SD)). All patients with OA were diagnosed on criteria developed by the American College of Rheumatology Diagnostic Subcommittee for OA [19]. At the time of surgery, the patients had symptomatic disease requiring medical treatment in the form of non-steroidal anti-inflammatory drugs or selective COX-2 inhibitors. Patients who had received intra-articular injections of steroids were excluded. The Clinical Research Ethics Committee of Notre-Dame Hospital approved the study protocol and the use of human tissues.

Chondrocytes were released from cartilage by sequential enzymatic digestion as described previously [11]. In brief, this consisted of 2 mg/ml pronase for 1 hour followed by 1 mg/ml collagenase for 6 hours (type IV; Sigma-Aldrich) at 37°C in DMEM and antibiotics (100 U/ml penicillin, 100 μg/ml streptomycin). The digested tissue was briefly centrifuged and the pellet was washed. The isolated chondrocytes were seeded at high density in tissue culture flasks and cultured in DMEM supplemented with 10% heat-inactivated FCS. At confluence, the chondrocytes were detached, seeded at high density, and allowed to grow in DMEM supplemented as above. The culture medium was changed every second day, and 24 hours before the experiment the cells were incubated in fresh medium containing 0.5% FCS. Only first-passaged chondrocytes were used.

### Immunohistochemistry

Cartilage specimens were processed for immunohistochemistry as described previously [4]. The specimens were fixed in 4% paraformaldehyde and embedded in paraffin. Sections (5 μm thick) of paraffin-embedded specimens were deparaffinized in toluene, then dehydrated in a graded ethanol series. The specimens were then preincubated with chondroitinase ABC (0.25 U/ml in PBS, pH 8.0) for 60 minutes at 37°C, followed by incubation with Triton X-100 (0.3%) for 30 minutes at 25°C. Slides were then washed in PBS followed by 2% hydrogen peroxide/methanol for 15 minutes. They were further incubated for 60 minutes with 2% normal serum (Vector Laboratories, Burlingame, CA, USA) and overlaid with primary antibody for 18 hours at 4°C in a humidified chamber. The antibody was a rabbit polyclonal anti-human PPARγ (Santa Cruz Biotechnology, Santa Cruz, CA, USA), used at 10 μg/ml. This antibody recognizes the epitope of the sequence mapping of amino acids 8 to 106 at the N terminus of PPARγ. Each slide was washed three times in PBS, pH 7.4, and stained with the use of the avidin-biotin complex method (Vectastain ABC kit; Vector Laboratories). The color was developed with 3,3'-diaminobenzidine (DAB) (Vector Laboratories) containing hydrogen peroxide. The slides were counterstained with eosin. The specificity of staining was evaluated by using antibody that had been preadsorbed (1 hour at 37°C) with a 20-fold molar excess of the protein fragment corresponding to amino acids 6 to 105 of human PPARγ (Santa Cruz), and by replacing the primary antibody with non-immune rabbit IgG (Chemicon, Temecula, CA, USA; used at the same concentration as the primary antibody). The evaluation of positive-staining chondrocytes was performed with our previously published method [4]. For each specimen, six microscopic fields were examined under ×40 magnification. The total number of chondrocytes and the number of positive-staining chondrocytes were evaluated and results were expressed as the percentage of chondrocytes that stained positive (cell score).

### RNA extraction and reverse transcriptase-polymerase chain reaction

Total RNA from homogenized cartilage or stimulated chondrocytes was isolated by using TRIzol^® ^reagent (Invitrogen) in accordance with the manufacturer's instructions. To remove contaminating DNA, isolated RNA was treated with RNase-free DNase I (Ambion, Austin, TX, USA). The RNA was quantified with the RiboGreen RNA quantitation kit (Molecular Probes, Eugene, OR, USA), dissolved in diethylpyrocarbonate-treated water and stored at -80°C until use. Total RNA (1 μg) was reverse-transcribed with Moloney murine leukemia virus reverse transcriptase (Fermentas, Burlington, ON, Canada) as detailed in the manufacturer's guidelines. One-fiftieth of the reverse transcriptase reaction was analyzed by real-time quantitative PCR as described below. The following primers were used: PPARγ1 sense, 5'-AAAGAAGCCAACACTAAACC-3'; PPARγ2 sense, 5'-GCGATTCCTTCACTGATAC-3'; common PPARγ1 and PPARγ2 antisense, 5'-CTTCCATTACGGAGAGATCC-3'; glyceraldehyde-3-phosphate dehydrogenase (GAPDH) sense, 5'-CAGAACATCATCCCTGCCTCT-3'; and GAPDH antisense, 5'-GCTTGACAAAGTGGTCGTTGAG-3'.

### Real-time quantitative PCR

Quantitative PCR analysis was performed in a total volume of 50 μl containing template DNA, 200 nM sense and antisense primers, 25 μl of SYBR^® ^Green master mix (Qiagen, Mississauga, ON, Canada) and uracil-*N*-glycosylase (UNG, 0.5 U; Epicentre Technologies, Madison, WI, USA). After incubation at 50°C for 2 minutes (UNG reaction), and at 95°C for 10 minutes (UNG inactivation and activation of the AmpliTaq Gold enzyme), the mixtures were subjected to 40 amplification cycles (15 s at 95°C for denaturation, and 1 minute for annealing and extension at 60°C). Incorporation of SYBR Green dye into PCR products was monitored in real time with a GeneAmp 5700 Sequence detection system (Applied Biosystems, Foster City, CA, USA) allowing determination of the threshold cycle (*C*_t_) at which exponential amplification of PCR products begins. After PCR, dissociation curves were generated with one peak, indicating the specificity of the amplification. A threshold cycle (*C*_t _value) was obtained from each amplification curve with the software provided by the manufacturer (Applied Biosystems).

Relative amounts of mRNA in normal and OA cartilage were determined with the use of the standard curve method. Serial dilutions of internal standards (plasmids containing cDNA of target genes) were included in each PCR run, and standard curves for the target gene and for GAPDH were generated by linear regression with a plot of log(*C*_t_) against log(cDNA relative dilution). *C*_t _values were then converted to the number of molecules. Relative mRNA expression in cultured chondrocytes was determined with the ΔΔ*C*_t _method, as detailed in the manufacturer's guidelines (Applied Biosystems). A Δ*C*_t _value was first calculated by subtracting the *C*_t _value for the housekeeping gene GAPDH from the *C*_t _value for each sample. A ΔΔ*C*_t _value was then calculated by subtracting the Δ*C*_t _value of the control (unstimulated cells) from the Δ*C*_t _value of each treatment. Fold changes compared with the control were then determined by raising 2 to the -ΔΔ*C*_t _power. Each PCR reaction generated only the expected specific amplicon as shown by the melting-temperature profiles of the final product and by gel electrophoresis of test PCR reactions. Each PCR was performed in triplicate on two separate occasions for each independent experiment.

### Plasmids and transient transfection

The luciferase reporter construct pGL3-PPARγ1p3000, containing a 3,000-base-pair fragment of the human PPARγ1 gene promoter, was kindly provided by Dr Johan Auwerx (Institut de Génétique et de Biologie Moléculaire et Cellulaire, Illkirch, France) [9]. β-Galactosidase reporter vector under the control of SV40 promoter (pSV40-β-galactosidase) was from Promega (Madison, WI, USA). Transient transfection experiments were performed with FuGene-6 (1 μg of DNA to 4 μl of FuGene 6; Roche Applied Science, Laval, QC, Canada) in accordance with the manufacturer's recommended protocol. In brief, chondrocytes were seeded and grown to 50 to 60% confluence. The cells were transfected with 1 μg of the reporter construct and 0.5 μg of the internal control pSV40-β-galactosidase. Six hours later, the medium was replaced with DMEM containing 1% FCS. The next day, the cells were treated for 18 hours with or without IL-1. After harvesting, luciferase activity was determined and normalized to β-galactosidase activity [16].

### Western blot analysis

Chondrocytes were lysed in ice-cold lysis buffer (50 mM Tris-HCl, pH 7.4, 150 mM NaCl, 2 mM EDTA, 1 mM PMSF, 10 μg/ml each of aprotinin, leupeptin, and pepstatin, 1% Nonidet P40, 1 mM Na_3_VO_4_, 1 mM NaF). Lysates were sonicated on ice and centrifuged at 12,000 r.p.m. for 15 minutes. The protein concentration of the supernatant was determined with the bicinchoninic acid method (Pierce, Rockford, IL, USA). Total cell lysate (20 μg) was subjected to SDS-PAGE and electrotransferred to a nitrocellulose membrane (Bio-Rad). After blocking in 20 mM Tris-HCl, pH 7.5, containing 150 mM NaCl, 0.1% Tween 20, and 5% (w/v) non-fat dry milk, blots were incubated overnight at 4°C with the primary antibody and washed with a Tris buffer (Tris-buffered saline, pH 7.5, containing 0.1% Tween 20). The blots were then incubated with horseradish peroxidase-conjugated secondary antibody (Pierce), washed again, incubated with SuperSignal Ultra Chemiluminescent reagent (Pierce), and, finally, exposed to Kodak X-Omat film (Eastman Kodak Ltd, Rochester, NY, USA).

### Statistical analysis

Data are expressed as means ± SEM unless stated otherwise. Statistical significance was assessed by the two-tailed Student's *t *test; *p *< 0.05 was considered significant.

## Results

### Decreased expression of PPARγ1 in OA cartilage

**Figure 1:**
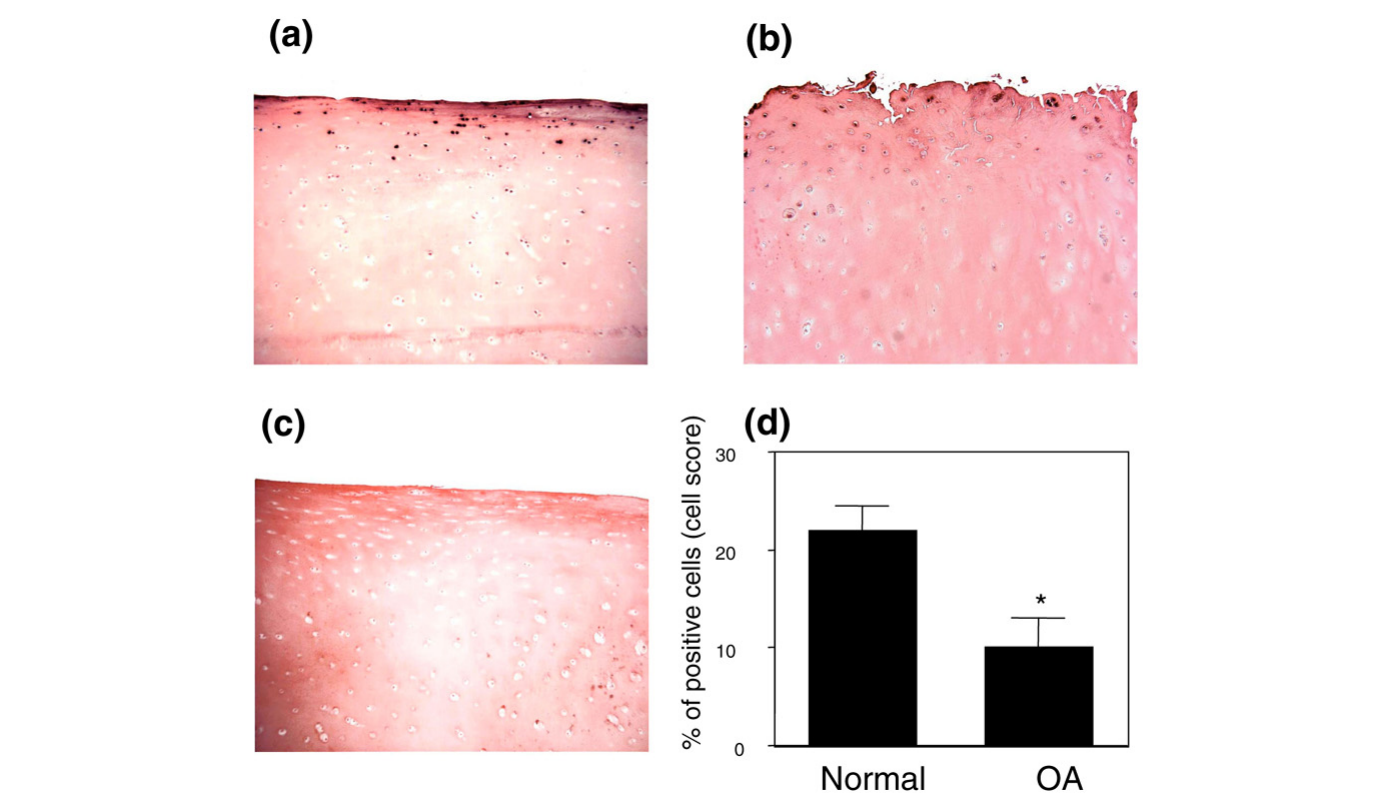
Expression of PPARγ protein in normal and osteoarthritis cartilage. Representative immunostaining of human normal cartilage **(a) **and cartilage from mild to moderate osteoarthritis (OA) **(b) **for peroxisome proliferator-activated receptor γ (PPARγ). **(c) **Normal specimens treated with anti-PPARγ antibody that was preadsorbed with a 20-fold molar excess of the protein fragment corresponding to amino acids 8 to 106 of human PPARγ (control for staining specificity). **(d) **Percentage of chondrocytes expressing PPARγ in normal and OA cartilage. The results are means ± SEM for 10 normal and 11 OA specimens. *p < 0.05 compared with normal cartilage.

To examine the expression and localization of PPAR-γ protein in cartilage, we performed an immunohistochemical analysis. We found that chondrocytes in both normal and OA cartilage express PPARγ protein. The immunostaining for PPARγ was essentially located in the superficial zones, and was lower in OA cartilage than in normal cartilage. Statistical evaluation of the cell score for PPARγ indicated significant differences between normal cartilage (22 ± 2.5% (mean ± SEM)) and cartilage from mild to moderate OA (11 ± 3%; Figure 1a,b). Similarly, PPARγ expression was significantly reduced in severe OA cartilage (10 ± 2%, data not shown). By contrast, in intact OA cartilage, the positive staining seemed lower, but the differences were not significant (data not shown). The specificity of the staining was confirmed by using antibodies that had been preadsorbed (1 hour, 37°C) with a 20-fold molar excess of the protein fragment corresponding to amino acids 6 to 105 of human PPARγ (Figure 1c) or non-immune serum (Figure 1c). PPARα and PPARβ were also expressed in normal, mild to moderate, and severe OA cartilage, but no significant differences were observed between the cartilage groups (Additional file 1).

**Figure 2:**
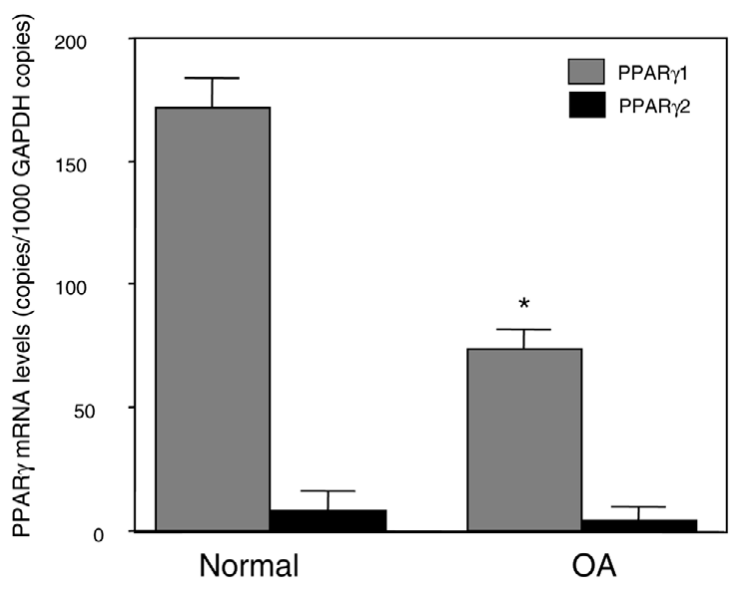
PPARγ1 and PPARγ2 mRNA levels in normal and osteoarthritis human cartilage. RNA was extracted from normal (*n *= 7) and osteoarthritis (*n *= 8) cartilage, reverse transcribed into cDNA, and processed for real-time PCR. The threshold cycle values were converted to the number of molecules, as described in the Materials and methods section. Data were expressed as copies of the gene's mRNA detected per 1,000 glyceraldehyde-3-phosphate dehydrogenase copies. *p < 0.05 compared with normal samples. PPAR, peroxisome proliferator-activated receptor.

PPARγ has two isoforms, PPARγ1 and PPARγ2, which are generated by alternative promoters and differential splicing [9]. To examine which PPARγ transcripts were expressed in cartilage, we determined absolute mRNA concentrations of PPARγ1 and PPARγ2 by quantitative real-time PCR. As shown in Figure 2, PPARγ1 abundance represents about 90% of the total PPARγ mRNA. Thus, human cartilage expresses high levels of γ1 mRNA, the isoform that is generally expressed in various tissues, and low levels of the γ2 isoform, which is more selectively expressed in adipose tissue [10]. The level of PPARγ1 expression in OA cartilage was 2.4-fold lower than in normal cartilage (*p *< 0.005). However, no significant differences in mRNA levels of PPARγ2 were seen between normal and OA cartilage (Figure 2). These observations demonstrate a selective downregulation of PPARγ1 in OA cartilage. In preliminary experiments we showed that the amplification efficiency of PPARγ1, PPARγ2, and GAPDH were approximately equal, ranging between 1.95 and 2.

### Time-course and dose-dependent effect of IL-1 on PPARγ1 expression in chondrocytes

**Figure 3:**
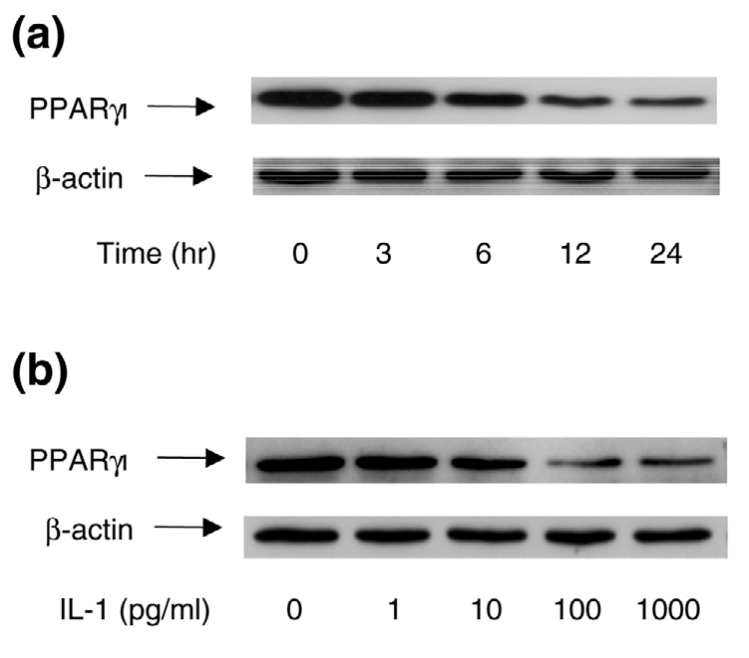
Effect of IL-1 on PPARγ1 protein expression in osteoarthritis chondrocytes. **(a) **Osteoarthritis (OA) chondrocytes were treated with 100 pg/ml IL-1 for the indicated periods. **(b) **OA chondrocytes were treated with increasing concentrations of IL-1 for 24 hours. Cell lysates were prepared and analyzed for peroxisome proliferator-activated receptor γ1 (PPARγ1) protein by Western blotting (upper panels). The blots were stripped and reprobed with a specific anti-β-actin antibody (lower panels). The blots are representative of similar results obtained from four independent experiments.

The reduced expression of PPARγ1 in OA cartilage suggests that humoral factors produced in the OA joint downregulate PPARγ1 expression. We therefore evaluated the effect of IL-1, one of the most prominent mediators in OA, on PPARγ1 expression in cultured chondrocytes. OA chondrocytes were treated with 100 pg/ml IL-1 for 0, 3, 6, 12, and 24 hours; the levels of PPARγ1 protein were then analyzed by Western blotting. In preliminary experiments we found that, as in cartilage, cultured chondrocytes express predominantly the PPARγ1 isoform but not the adipocyte-specific PPARγ2 isoform. As shown in Figure 3a, PPARγ1 protein expression was not significantly affected after 3 hours of stimulation with IL-1. The level of PPARγ1 protein then started to decline gradually at 6 hours and remained low until at least 24 hours. Subsequently, we examined the effect of various concentrations of IL-1 on PPARγ1 protein expression. As shown in Figure 3b, the expression of PPARγ1 was downregulated by IL-1 in a concentration-dependent manner; significant decreases were observed at a concentration as low as 10 pg/ml. Maximal decreases were obtained at an IL-1 concentration of 100 pg/ml (Figure 3b). No modulation of PPARα and PPARβ expression was seen (Additional file 2).

**Figure 4:**
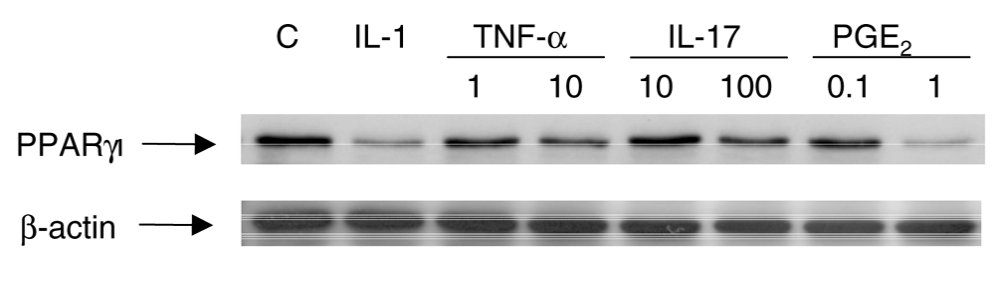
Effect of TNF-α, IL-17 and prostaglandin E_2 _on PPARγ1 protein expression in osteoarthritis chondrocytes. Cells were treated with IL-1 (100 pg/ml), TNF-α (1 and 10 ng/ml), IL-17 (10 and 100 ng/ml), and prostaglandin E_2 _(0.1 and 1 μM). After 24 hours, cell lysates were prepared and analyzed for peroxisome proliferator-activated receptor γ1 (PPARγ1) protein expression by Western blotting. In the lower panel, the blots were stripped and reprobed with a specific anti-β-actin antibody. The blots are representative of similar results obtained from four independent experiments.

In addition to IL-1, the pro-inflammatory mediators TNF-α, IL-17, and PGE_2 _also contribute to the pathogenesis of OA [1-3]. We therefore examined their effects on PPARγ1 protein expression. Cultured OA chondrocytes were incubated for 24 hours with IL-1 (100 pg/ml), TNF-α (1 and 10 ng/ml), IL-17 (10 and 100 ng/ml), and PGE_2 _(0.1 and 1 μM), and the expression levels of PPARγ1 were determined by Western blotting. As shown in Figure 4, and like IL-1, TNF-α, IL-17, and PGE_2 _also downregulated PPARγ1 protein expression. Similar results were obtained with normal chondrocytes (*n *= 3; data not shown).

### Downregulation by IL-1 of PPARγ1 expression at the transcriptional level

**Figure 5:**
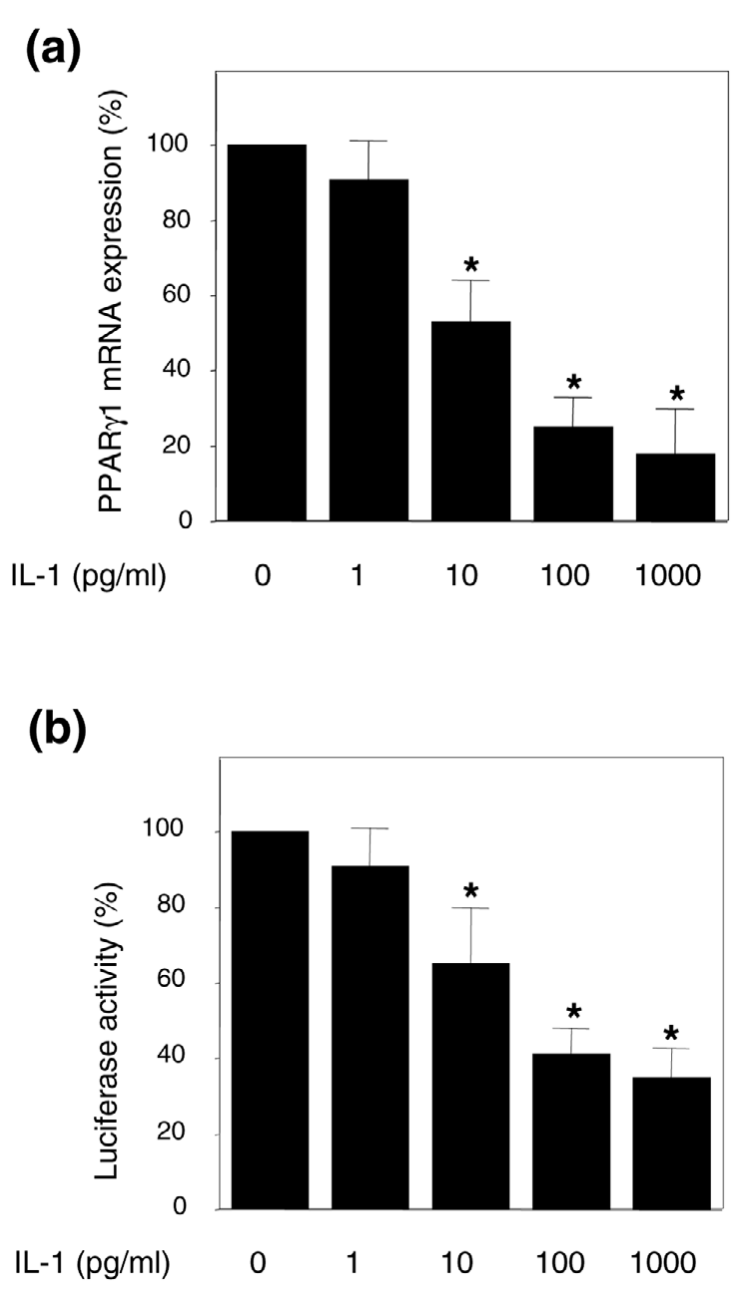
IL-1 downregulates PPARγ1 expression at the transcriptional level. **(a) **Osteoarthritis (OA) chondrocytes were treated with increasing concentrations of IL-1 for 12 hours. Total RNA was isolated and reverse transcribed into cDNA, and peroxisome proliferator-activated receptor γ1 (PPARγ1) and glyceraldehyde-3-phosphate dehydrogenase mRNAs were quantified by real-time PCR. All experiments were performed in triplicate, and negative controls without template RNA were included in each experiment. **(b) **OA chondrocytes were co-transfected with 1 μg per well of the PPARγ1 promoter (pGL3-PPARγ1p3000) and 0.5 μg per well of the internal control pSV40-β-galactosidase, using FuGene 6 transfection reagent. The next day, transfected cells were treated with increasing concentrations of IL-1 for 18 hours. Luciferase activity values were determined and normalized to β-galactosidase activity. Results are expressed as percentage changes, taking the value of untreated cells as 100%, and show means ± SEM for four independent experiments. *p < 0.05 compared with untreated cells.

To elucidate the mechanism responsible for the changes in amounts of PPARγ1 protein, we measured the steady-state level of PPARγ1 mRNA by quantitative real-time PCR. Expression of the gene encoding GAPDH was used for normalization. The relative expression level of PPARγ1 mRNA was plotted as a percentage decrease compared with untreated control cells (Figure 5a). Consistent with its effects on protein expression (Figure 3b), IL-1 downregulates PPARγ1 mRNA expression in a dose-dependent manner in OA chondrocytes. The effect of IL-1 on PPARγ1 mRNA expression was maximal (about 85% decrease) at 100 pg/ml. A dose-dependent effect of IL-1 on PPARγ1 mRNA expression was also observed in normal chondrocytes (*n *= 3; data not shown).

To characterize the effect of IL-1 on PPARγ1 expression further, we performed transient transfection experiments with the reporter construct pGL3-PPARγ1p3000, containing about 3,000 base pairs of regulatory sequence of the gene encoding human PPARγ1 [9]. As shown in Figure 5b, IL-1 suppressed PPARγ1 promoter activity in a dose-dependent manner. The effect of IL-1 on PPARγ1 promoter activity was optimal at 100 pg/ml (about 65% decrease). Taken together, these data strongly suggest that IL-1 suppressed PPARγ1 expression at the transcriptional level.

### The MAPKs JNK and p38, but not ERK, are involved in IL-1-induced downregulation of PPARγ1

**Figure 6:**
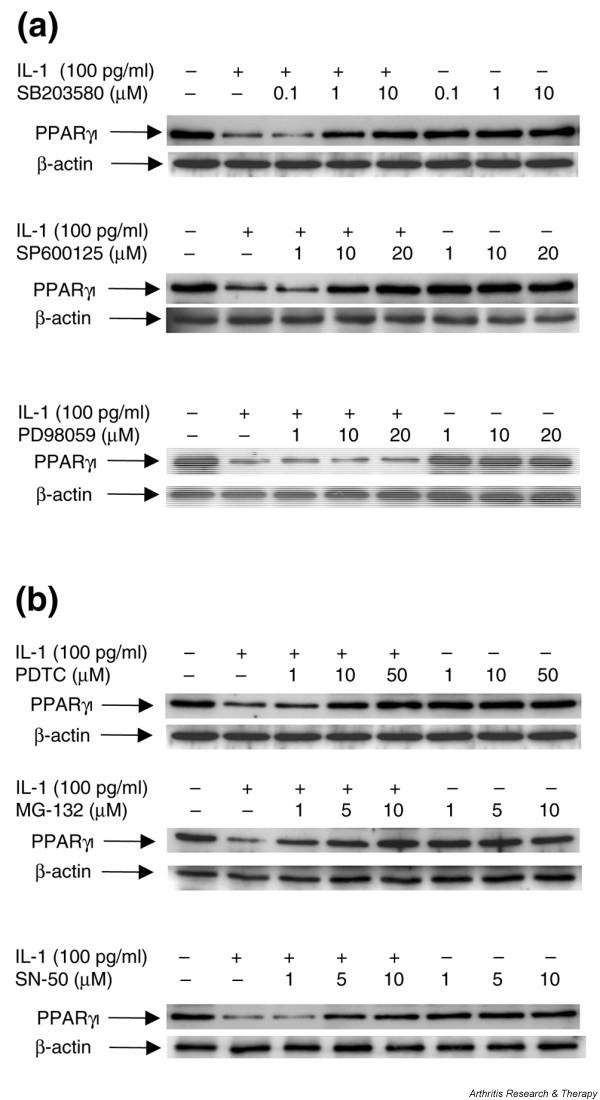
Effect of mitogen-activated protein kinase and NF-κB inhibitors on IL-1-induced downregulation of PPARγ1 expression. **(a) **Osteoarthritis (OA) chondrocytes were exposed to increasing concentrations of SB203580 (p38 mitogen-activated protein kinase inhibitor), SP600125 (c-Jun N-terminal kinase inhibitor) and PD98059 (extracellular signal-regulated kinase inhibitor) for 30 minutes before treatment with or without IL-1 (100 pg/ml). **(b) **OA chondrocytes were exposed to increasing concentrations of various inhibitors of NF-κB (pyrrolidine dithiocarbamate, MG-132, and SN-50) for 30 minutes before stimulation with or without IL-1 (100 pg/ml). After 24 hours, cell lysates were prepared and analyzed for peroxisome proliferator-activated receptor γ1 (PPARγ1) protein expression by Western blotting. In the lower panels, the blots were stripped and reprobed with a specific anti-β-actin antibody. The blots are representative of similar results obtained from four independent experiments.

IL-1 is known to induce its effects in chondrocytes through activation of a plethora of signaling pathways, including the mitogen-activated protein kinases (MAPKs) c-Jun N-terminal kinase (JNK), p38, and extracellular signal-regulated kinase (ERK) [20]. To assess the contribution of these pathways in the IL-1-mediated downregulation of PPARγ1, OA chondrocytes were pretreated for 30 minutes with selective inhibitors for the above pathways, and then stimulated or not with IL-1 for 18 hours. Total cell lysates were analyzed for PPARγ1 protein expression by Western blotting. As shown in Figure 6a, IL-1 reduced PPARγ1 expression remarkably, confirming the results seen previously (Figure 3). Pretreatment with SB203580, a specific p38 MAPK inhibitor, as well as pretreatment with SP600125, a selective inhibitor of JNK, dose-dependently abolished IL-1-induced downregulation of PPARγ1 expression. Conversely, PD98059, a selective inhibitor of ERK, had no effect on IL-1-induced downregulation of PPARγ expression, even at a high concentration (20 μM). None of the MAPK inhibitors had an effect on PPARγ expression in the absence of IL-1. These results suggest that the MAPKs JNK and p38, but not ERK, are involved in the suppression of PPARγ1 expression by IL-1.

### Mediation of IL-1-induced downregulation of PPARγ1 by NF-κB

Because NF-κB mediates many of the effects of IL-1 in a variety of cell types including chondrocytes, we examined the role of this transcription factor in the repression of PPARγ1. We used three different pharmacological inhibitors of the NF-κB pathway: the antioxidant PDTC, a proteasome inhibitor MG-132, and an inhibitor of NF-κB translocation (SN-50). Cells were pretreated with increasing concentrations of each inhibitor for 30 minutes and then subsequently treated with 100 pg of IL-1 for 18 hours.

As shown in Figure 6b, treatment with IL-1 decreased PPARγ1 expression, but this IL-1 effect was dose-dependently abolished in the presence of each of the three NF-κB inhibitors (PDTC, MG-132, and SN-50). None of the NF-κB inhibitors had an effect on basal PPARγ1 expression. These results imply that NF-κB activation participates in the IL-1-mediated downregulation of PPARγ1 expression.

## Discussion

There is considerable evidence for the importance of PPARγ in OA because of its potential beneficial effects. It is expressed by all major cells in joints, including chondrocytes [11,13]. Natural and synthetic ligands of PPARγ were shown to inhibit the expression of several inflammatory and catabolic genes in cultured chondrocytes [4,11,12] and to exhibit anti-inflammatory and chondroprotective effects in an experimental animal model of OA [18]. However, little is known about the expression and regulation of PPARγ expression in cartilage. Here, we analyzed the expression of PPARγ in OA and normal cartilage, and studied the effect of IL-1, a prominent cytokine in OA, on PPARγ expression in cultured chondrocytes.

This is the first study to demonstrate that human cartilage expresses predominantly PPARγ1 mRNA and that the levels of PPARγ1 are decreased in OA in comparison with normal cartilage. Our immunohistochemistry analysis showed that PPARγ was located essentially in the superficial zone of cartilage and that the levels of PPARγ expression in OA cartilage were lower than in normal cartilage.

Altered expression of PPARγ was observed in several other inflammatory disorders. For instance, PPARγ expression was shown to be reduced in atherosclerotic tissues [21], in epithelial cells from patients with ulcerative colitis [22], in peripheral blood mononuclear cells from patients with multiple sclerosis [23], in alveolar macrophages from patients with allergic asthma [24], and in nasal polyposis from patients with allergic rhinitis [25]. In contrast, PPARγ expression was shown to be elevated in brains of patients with Alzheimer's disease [26], in bronchial epithelium and airway smooth muscle cells of asthmatic patients [27], and in T cells isolated from patients with sepsis [28]. Taken together, these results suggest that tissue-specific regulation of PPARγ expression is extremely complex.

To determine which factors might downregulate PPARγ expression in cartilage, we tested the impact of IL-1, which accumulates in chondrocytes in the superficial zone of OA cartilage [29,30] and has a pivotal role in the initiation and progression of OA [1-3]. Our results revealed that exposure to IL-1 downregulates PPARγ protein expression in chondrocytes in a time- and dose-dependent manner. It should be noted that TNF-α, IL-17, and PGE_2_, which are known to contribute to the pathogenesis of OA, also downregulate PPARγ gene expression. We therefore cannot exclude the possibility of a role for these inflammatory mediators in PPARγ downregulation in cartilage *in vivo*. Given the anti-inflammatory and anti-catabolic functions of PPARγ, it is reasonable to speculate that the suppression of PPARγ expression by inflammatory mediators in chondrocytes presents a new and additional mechanism by which these mediators contribute to the pathogenesis of OA. Our findings are consistent with other studies showing that pro-inflammatory stimuli downregulate PPARγ expression in chondrocytes [31-33] and synovial fibroblasts [34,35]. In contrast, Shan and colleagues [36] found that IL-1 upregulates PPARγ expression in chondrocytes. The reasons for these discrepancies are not clear and could be due to small differences in chondrocyte preparation, culture conditions, and/or detection methods.

Suppression of PPARγ1 expression by IL-1 in chondrocytes probably occurs at the transcriptional level, because reporter gene assays revealed a decrease in PPARγ1 promoter activity by IL-1. As an alternative to an effect on PPARγ1 promoter, we could not exclude a specific effect of IL-1 on the stability of PPARγ1 mRNA.

The MAPKs JNK, p38, and ERK are activated by IL-1 and mediate many of the effects of IL-1 in chondrocytes [20]. To determine whether these MAPKs are involved in the IL-1-mediated downregulation of PPARγ1 expression, we employed specific inhibitors of the three MAPKs. We found that SB203580 and SP600125 – specific inhibitors of the MAPKs p38 and JNK, respectively – almost completely abolished the IL-1-mediated downregulation of PPARγ1 expression, whereas PD98059 – an inhibitor of the MAPK ERK- was without effect. These data suggest that the MAPKs JNK and p38, but not ERK, mediate IL-1-induced downregulation of PPARγ1 expression in chondrocytes. The NF-κB pathway also mediates many effects of IL-1 in chondrocytes [37-41]. We demonstrate here that three compounds that interfere with NF-κB activation, the anti-oxidant PDTC, the proteasome inhibitor MG-132, and an inhibitor of NF-κB translocation SN-50, blocked the suppressive effect of IL-1, suggesting the involvement of NF-κB in the IL-1-mediated downregulation of PPARγ1 in chondrocytes. Thus, IL-1 engages both the MAPK (JNK and p38) and the NF-κB pathways to suppress PPARγ1 expression, although it is not clear whether these pathways act on the same axis or in parallel. Downstream nuclear events in JNK, p38, and NF-κB signaling pathways leading to the regulation of gene expression in chondrocytes include the activation of the transcription factors AP-1 and NF-κB [20,37,38,40-43]. The human PPARγ1 promoter contains binding sites for both AP-1 and NF-κB [9]. It is therefore possible that AP-1 and NF-κB mediate IL-1-induced downregulation of PPARγ1 expression. Although they are historically characterized as transcriptional activators, several reports have recently defined AP-1 and NF-κB as transcriptional repressors [44-50]. Analysis of PPARγ1 promoter in a promoter reporter construct, with mutation of the AP-1 and NF-κB response elements and the use of small interfering RNA technology, will contribute to our understanding of the importance of AP-1 and NF-κB in the IL-1-induced downregulation of PPARγ1 expression.

The physiological significance of reduced expression of PPARγ in OA cartilage is of considerable interest, given the protective functions of PPARγ in cartilage. Indeed, we and others have previously reported that PPARγ activators inhibit several inflammatory and catabolic events involved in the pathogenesis of OA [4,11,12,32-34]. PPARγ activation was also shown to prevent the proteoglycan degradation induced by pro-inflammatory cytokines [13]. Furthermore, PPARγ ligands were shown to reduce the incidence and severity of OA in an experimental model, preventing inflammatory and catabolic responses as well as cartilage degradation [18]. All these data suggest that PPARγ has a protective role in OA. This is strengthened by the observation that PPARγ haploinsufficiency exacerbates experimentally induced arthritis [51]. It is therefore tempting to speculate that diminished expression of PPARγ in OA cartilage may, at least in part, be involved in increased expression of inflammatory and catabolic genes, promoting articular inflammation and cartilage degradation. In addition, the observation that IL-1 and other pro-inflammatory mediators downregulate PPARγ1 expression in chondrocytes has important implications for our understanding of the pathophysiology of OA.

## Conclusion

The decreased expression of PPARγ in OA cartilage and the literature supporting a protective role for PPARγ in OA raise the possibility that upregulation of PPARγ may be beneficial in the context of preventing and treating OA. Additional studies to define the molecular mechanisms controlling the expression of PPARγ are therefore urgently needed. Such research will no doubt add to our understanding of the pathogenesis of OA, and could lead to the development of new therapeutic strategies in the prevention and treatment of OA and possibly other arthritic diseases.

## Abbreviations

AP-1 = activator protein 1; COX = cyclooxygenase; DMEM = Dulbecco's modified Eagle's medium; ERK – extracellular signal-regulated kinase; FCS = fetal calf serum; GAPDH = glyceraldehyde-3-phosphate dehydrogenase; IL = interleukin; JNK = c-Jun N-terminal kinase; MAPK = mitogen-activated protein kinase; MMP = metalloproteinase; mPGES = membrane-associated prostaglandin E synthase; NF-κB = nuclear factor-κB; OA = osteoarthritis; PDTC = pyrrolidine dithiocarbamate; PG = prostaglandin; PGE_2 _= prostaglandin E_2_; PPAR = peroxisome proliferator-activated receptor; RT-PCR = reverse-transcriptase-mediated polymerase chain reaction; TNF = tumor necrosis factor.

## Competing interests

The authors declare that they have no competing interests.

## Authors' contributions

HA conceived the study, designed and performed cell and real-time RT-PCR experiments and some immunohistochemistry experiments. MB participated in the study design and data analysis. LM-E performed some immunohistochemistry experiments. JM-P, J-PP, and ND helped to obtain tissues, participated in some immunohistochemistry studies and gave critical comments on the manuscripts. HF conceived, designed, and coordinated the study, performed some cell experiments, and drafted the manuscript. All authors read and approved the final manuscript.

## Supplementary Material

Additional file 1 A PDF file showing the expression of PPARα and PPARβ proteins in normal and OA cartilage. Click here for file

Additional file 2 A PowerPoint file showing the effect of IL-1 on PPARα and PPARβ protein expression in OA chondrocytes. Click here for file
