# Supplementary figures and images for: Peroxisome proliferator-activated receptor γ1 expression is diminished in human osteoarthritic cartilage and is downregulated by interleukin-1β in articular chondrocytes
Source: Arthritis Res Ther. 2007 Mar 26;9(2):R31. doi: 10.1186/ar2151 (PMC1906809; doi:10.1186/ar2151)

Supplemental file 1

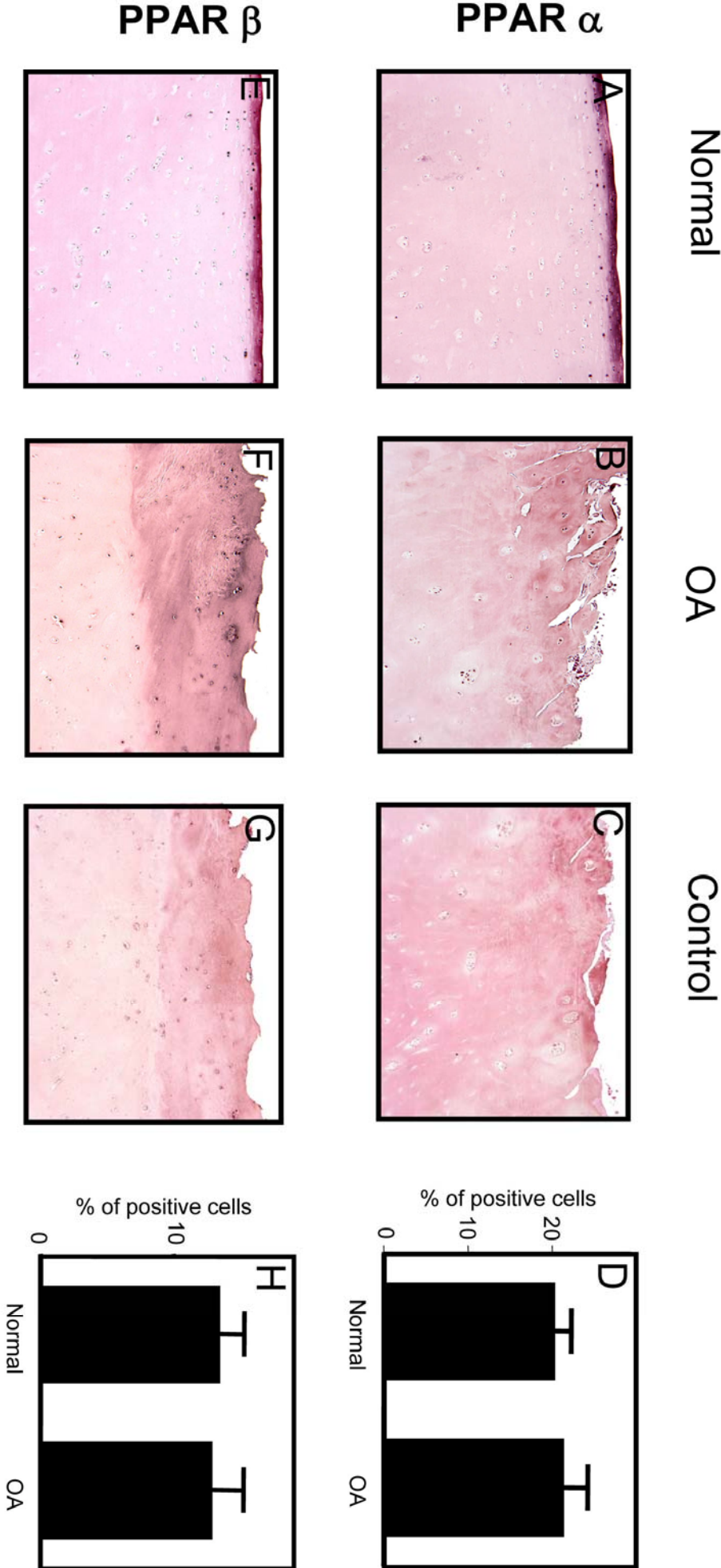

Supplement: Additional file 1 — A PDF file showing the expression of PPARα and PPARβ proteins in normal and OA cartilage. [file ar2151-S1.pdf]
