# Supplementary material for: Peroxisome proliferator-activated receptor γ1 expression is diminished in human osteoarthritic cartilage and is downregulated by interleukin-1β in articular chondrocytes
Source: Arthritis Res Ther. 2007 Mar 26;9(2):R31. doi: 10.1186/ar2151 (PMC1906809; doi:10.1186/ar2151)
Supplement: Additional file 2 — A PowerPoint file showing the effect of IL-1 on PPARα and PPARβ protein expression in OA chondrocytes. [file ar2151-S2.ppt]

## Slide 1
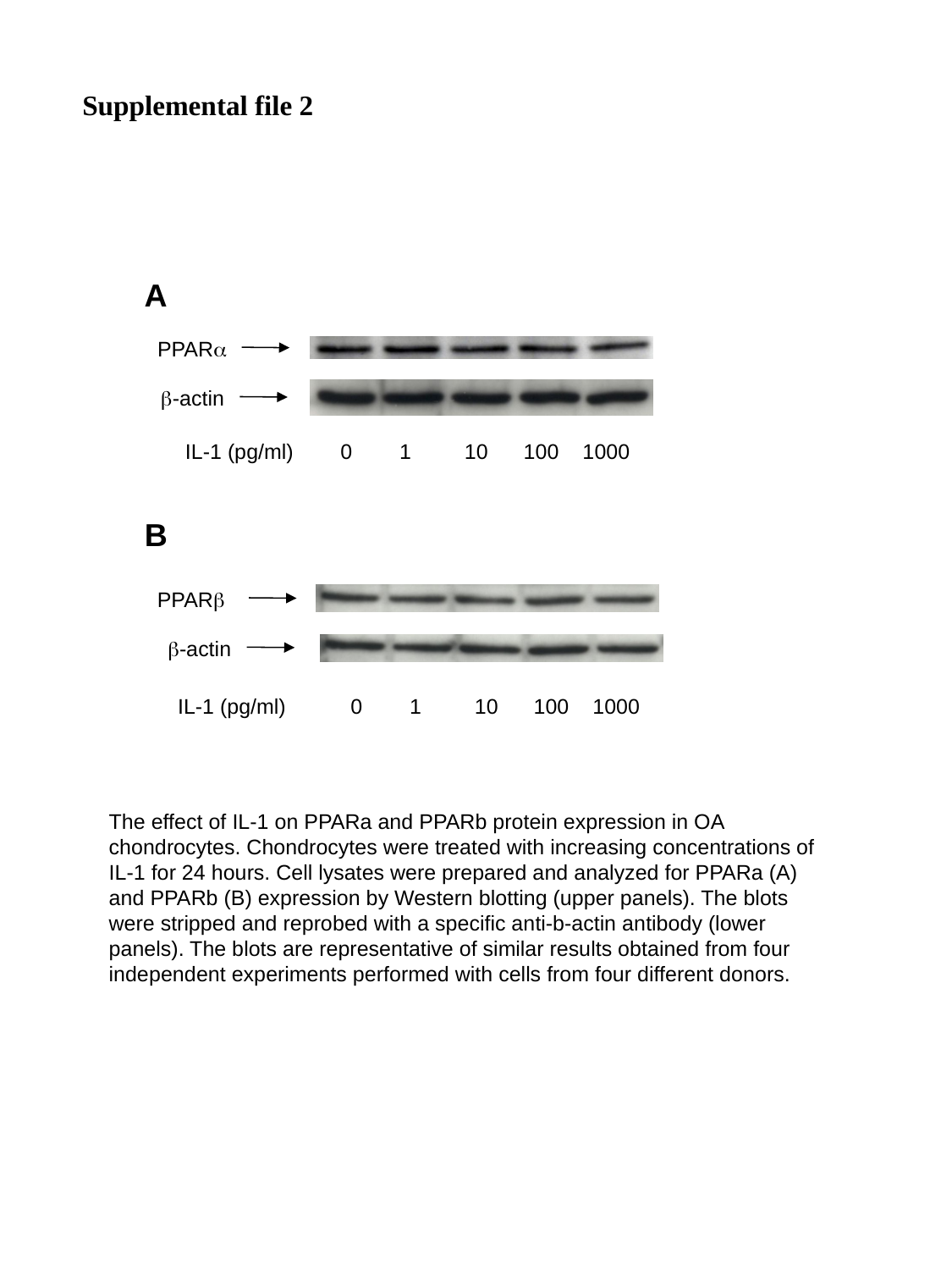

Supplemental file 2
A
PPARα
β-actin
IL-1 (pg/ml) 0 1 10 100 1000
B
PPARβ
β-actin
IL-1 (pg/ml) 0 1 10 100 1000
The effect of IL-1 on PPARa and PPARb protein expression in OA chondrocytes. Chondrocytes were treated with increasing concentrations of IL-1 for 24 hours. Cell lysates were prepared and analyzed for PPARa (A) and PPARb (B) expression by Western blotting (upper panels). The blots were stripped and reprobed with a specific anti-b-actin antibody (lower panels). The blots are representative of similar results obtained from four independent experiments performed with cells from four different donors.
